# Early Clinical Features, Time to Secondary Progression, and Disability Milestones in Polish Multiple Sclerosis Patients

**DOI:** 10.3390/medicina55060232

**Published:** 2019-05-31

**Authors:** Łukasz Rzepiński, Monika Zawadka-Kunikowska, Zdzisław Maciejek, Julia L. Newton, Paweł Zalewski

**Affiliations:** 1 Department of Neurology, 10th Military Research Hospital and Polyclinic, 85-681 Bydgoszcz, Poland; z.maciejek@wp.pl; 2 Department of Hygiene, Epidemiology and Ergonomy, Nicolaus Copernicus University, Torun, 85-094 Bydgoszcz, Poland; monikazawadka@poczta.onet.pl (M.Z.-K.); p.zalewski@cm.umk.pl (P.Z.); 3 Institute of Cellular Medicine, Newcastle University, Framlington Place, Newcastle Upon Tyne NE2 4HH, UK; julia.newton@ncl.ac.uk

**Keywords:** multiple sclerosis, disability, conversion, Poland

## Abstract

*Background and Objectives:* Determining the clinical course of multiple sclerosis (MS) and prediction of long-term disability can be a big challenge. To determine early clinical features of MS, their influence on long-term disability progression, and time to transition from relapsing-remitting MS (RRMS) to secondary progressive MS (SPMS), a cohort of Polish patients was studied. *Materials and Methods:* We retrospectively evaluated 375 Polish MS patients based on data from available medical records. We assessed early clinical MS features and the relationship between demographics and time from disease onset to attainment of 4 and 6 points on the Expanded Disability Status Scale (EDSS), as well as time to conversion from RRMS to SPMS. *Results:* The differences between initial MS variants were significantly associated with gender, age at disease onset, number and type of the first symptoms, and rate of the disability accrual. Mean times from disease onset to attainment of EDSS 4 and 6 were significantly influenced by the disease variant, age at onset, gender, degree of recovery from the initial symptoms, and first inter-bouts interval. The mean time to secondary progression was significantly influenced by the number and type of the first symptoms of RRMS. *Conclusions:* Early clinical features of MS are important in determining the disease variant, the time to transition from RRMS to SPMS, as well as predicting the disability accumulation of patients. Despite the small differences regarding the first MS symptoms, the disability outcomes in the cohort of Polish patients are similar to other regions of the world.

## 1. Introduction

Multiple sclerosis (MS) is characterised by two clinical phenomena: relapses and disability progression [1,2,3].

Relapsing-remitting multiple sclerosis (RRMS) is considered to be a two-stage disease with an early focal inflammation phase followed by progression characterized by neurodegeneration. In primary progressive multiple sclerosis (PPMS), the predominant mechanisms of neurodegeneration are observed from the onset of the disease [4,5,6,7].

Disability accumulation in patients with relapsing-remitting multiple sclerosis (RRMS) may result from incomplete recoveries from relapses, development of secondary progressive MS (SPMS), or a combination of these two factors [8,9,10,11]. Numerous reports suggest that PPMS is associated with a worse prognosis than RRMS or secondary progressive MS [5,12,13,14]. Earlier studies on the natural history of multiple sclerosis have indicated some demographic and early clinical features that can predict the progression of irreversible disability [10,15]. However, data according to the relationship between initial MS symptoms and disability progression, as well as the time to transition from RRMS to SPMS, have been inconsistent. In the age of widespread availability of therapies modifying the course of MS, the analysis of the impact of early clinical features on the disability accrual is rare. There are no data on the relationships between early clinical features and long-term disability outcomes in Polish MS patients. 

The aim of the study was to determine the early clinical features of multiple sclerosis, their influence on disability progression and time to transition from RRMS to SPMS in a cohort of Polish patients. 

## 2. Materials and Methods

### 2.1. Patients and Data Collection

We conducted a single-center analysis of 375 Polish MS patients hospitalized at the Department of Neurology in northern Poland between 30 June 2008 and 31 October 2016. The inclusion criteria were that patients had multiple sclerosis diagnosed in accordance with the diagnostic criteria in force at the time of the diagnosis and that patients were at least 18 years old.

Demographic and clinical data, as well as the type of treatment applied, were collected by retrospective analysis of medical records from our database. Because of a significantly wide span of disease duration in the studied group, all diagnostic criteria developed up to 2010 were used for the establishing of the diagnosis [16,17,18,19]. The first manifestations of the disease were divided into mono- and polysymptomatic forms (≥2 symptoms). The isolated initial symptoms included motor deficit, sensory symptoms, optic neuritis, balance disturbances, vertigo, double vision, cranial nerve dysfunction, gait disturbances, sphincter dysfunctions, coordination impairment, and neuralgias. The disease duration was defined as the time from the manifestation of its first signs. In the study, the following clinical variants of the disease were taken into account: relapsing-remitting (RRMS), secondary progressive (SPMS), and primary progressive (PPMS). RRMS was characterized by disease bouts followed by a complete or partial recovery, without progression of disability between the relapses. A relapse was defined as the occurrence of new or the worsening of previous MS-related symptoms lasting over 24 hours and unrelated to fever or infection. Symptoms occurring within one month were considered as a single bout. Residual symptoms from the first bout were defined as the persistence of neurological deficit, corresponding to an Expanded Disability Status Scale (EDSS) score ≥2 [4]. SPMS was diagnosed in RRMS patients after confirming the insidious and irreversible deterioration of neurological deficits of ≥6 months, regardless of the superimposed relapses [1]. PPMS was defined as at least one-year deterioration of neurological symptoms from the disease onset with or without superimposed episodes of worsening [1,10]. When the age of the first manifestation of MS was considered, the early-onset (EOMS, 16 years or less), the intermediate onset (IOMS, between 17 and 49 years) and the late-onset (LOMS, 50 years or more) was distinguished. 

Disability of patients was assessed according to the Kurtzke Expanded Disability Status Scale (EDSS) [20]. On the basis of this scale, groups of patients with mild (EDSS ≤ 3.5), moderate (4.0 ≤ EDSS ≤ 5.5), and severe (EDSS ≥ 6.0) levels of disability were distinguished. The progression of disability was determined by analyzing the time from MS onset to attainment of EDSS 4 and 6. We assessed the relationships between time from disease onset to reach an EDSS score of 4 and 6 and the clinical variant of MS, gender, initial symptoms, recovery from first relapse, interval between the first and second relapses, and cumulative bouts number in the first two and five years of the disease. All patients gave their informed consent to having their data stored in the database. This study was approved by the Ethical Committee of Ludwik Rydygier Collegium Medicum (protocol no. KB 473/2014, approved on 26 June 2014).

### 2.2. Stastistical Analysis

The normality of the distribution of variables was verified with the Shapiro–Wilk test. For samples with the distribution similar to normal, an arithmetic mean and a standard deviation were calculated, and means were compared with the Student’s *t*-test for independent variables. The Levene’s test was used to evaluate variance uniformity. When the distribution differed significantly from the normal distribution, the median was calculated, and the significance of differences between groups was verified with the non-parametric Mann–Whitney U-test. The chi-square (χ^2^) test was used to compare proportion in the groups. The survival function was estimated using the Kaplan–Meier function. Two survival curves were compared with the log-rank test. To examine correlations between variables, the Spearman’s correlation coefficient was used. The level of *p* = 0.05 was assumed as a statistical significance limit. All calculations were performed with Statistica 12.1 application.

## 3. Results

### 3.1. Population Characteristics

The demographic and clinical characteristics of the cohort are presented in Table 1 and Table 2. In the group of 375 patients, the gender ratio of females to males was 2.3:1, and the mean age was 43.1 ± 12.5 years. The median disease duration was 9.0 years. The mean age at the disease onset was 32.3 ± 10.9 years (ranging from 13 to 61 years). Of the patients, 13 (3.5%) were distributed to EOMS, 333 (88.8%) patients to IOMS, and 29 (7.7%) patients to LOMS with respect to age of the disease onset. No statistically significant differences were observed for the age of MS onset (*p* = 0.158) and the median of disease duration (*p* = 0.308) between groups of women and men. In all patients with EOMS, the initial disease course followed the RRMS pattern, while PPMS predominated in the group of the patients with LOMS (17/29, 58.6%) (*p* < 0.0001).

A total of 125 patients (33.3%) received immunomodulatory drugs (IMDs): interferon-beta 1a, interferon-beta 1b, glatiramer acetate, and peginterferon. The median time from the diagnosis to initiation of the IMDs was 1.0 year. The median duration of treatment with the IMDs was 3.0 years.

### 3.2. Clinical Features

The PPMS course was statistically more common in men than in women, while the initial RRMS course was statistically more frequent in women than in men (*p* < 0.001). The SPMS patients had significantly higher values for the median disease duration (15.0 years), when compared with the RRMS (6.0 years) and the PPMS (8.5 years) patients (*p* < 0.001). No statistically significant difference was found between the RRMS and PPMS patients for the median disease duration (*p* = 0.1160). The mean age of the patients at the moment of the manifestation of first symptoms was significantly higher in the PPMS group versus RRMS (41.2 ± 10.8 vs. 30.7 ± 10.2 years, *p* < 0.001), without statistically significant differences between women and men.

The number of initial symptoms statistically significantly depended on the clinical disease variant. The monosymptomatic disease onset was more frequent in the RRMS patients (217, 68.2%), while the polysymptomatic disease onset was more common in the PPMS patients (34, 59.6%) (*p* < 0.001). Amongst the 135 (36%) subjects with polysymptomatic disease onset, two symptoms occurred in 96 (25.3%), three in 32 (8.5%), and four in 8 (2.1%) patients.

In the study cohort, the mean time to conversion from RRMS to SPMS was 12.7 ± 7.4 years (a range of 0.5–42 years) without statistically significant differences between women and men (13.2 ± 7.9 vs. 11.9 ± 6.6 years; *p* = 0.3848). The observed ratio of the patients with RRMS conversion to SPMS was higher for men than for women; however, that difference was not significant statistically (*p* = 0.127). The monosymptomatic RRMS onset was associated with late conversion to SPMS, while the polysymptomatic disease onset was related to earlier conversion to SPMS (14.2 ± 7.7 years; 10.1 ± 6.2 years, respectively; *p* = 0.006). The first clinical manifestation in the form of a motor deficit was associated with a faster conversion to SPMS (*p* < 0.001), while the disease onset in the form of optic neuritis was associated with a later conversion to SPMS (*p* = 0.002). A shorter time of conversion to SPMS was also noted for initial manifestations of the disease with sphincter dysfunctions and coordination impairment; however, the differences found were not statistically significant (Table 3).

### 3.3. Disability

**Figure 1:**
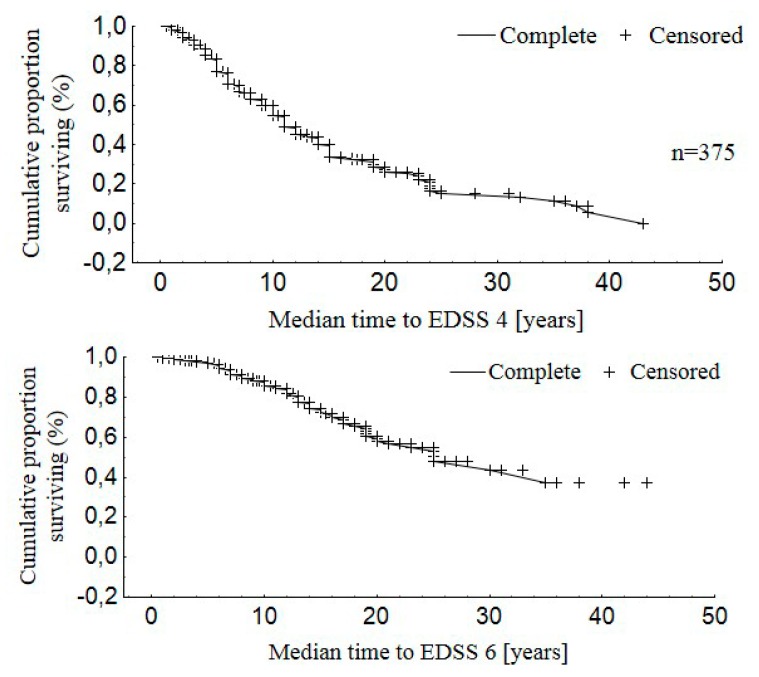
Distribution of the time to disability equal to 4 and 6 points on the EDSS scale for the whole studied group.

**Figure 2:**
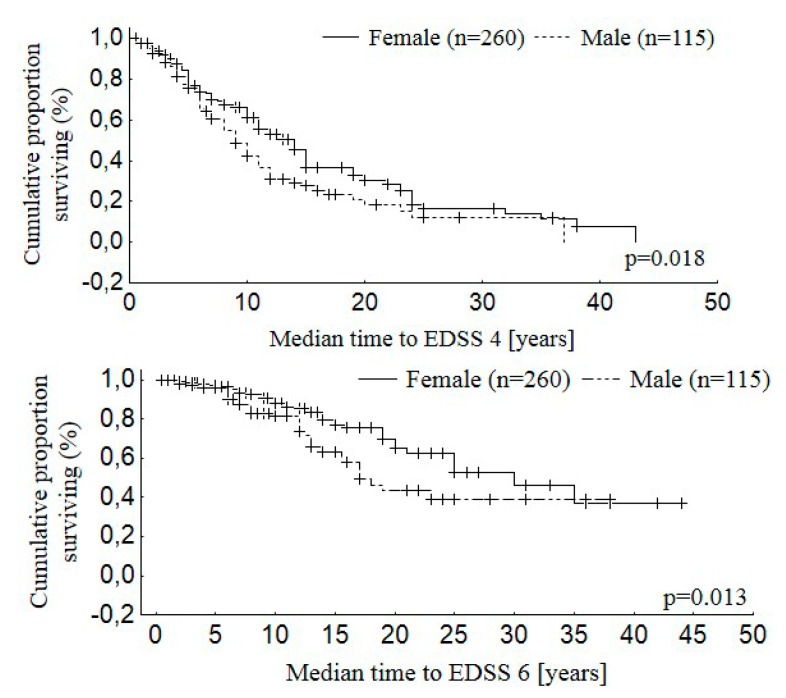
Distribution of the time to disability equal to 4 and 6 points on the EDSS scale for females and males.

**Figure 3:**
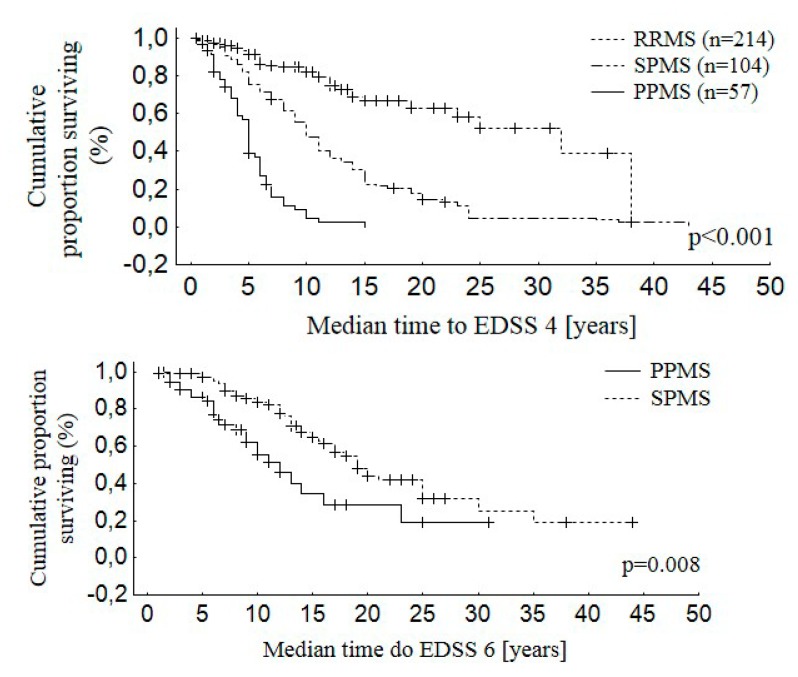
Distribution of the time to disability equal to 4 and 6 points on the EDSS scale for RRMS, PPMS, and SPMS.

Disability of patients evaluated on the EDSS scale ranged from 1 to 8 points. The mean EDSS score was statistically significantly lower in the group of women versus men (*p* = 0.001). Patients’ disability assessed on the EDSS scale after the first five years of the disease was 2.1 ± 1.1 points for RRMS and 4.1 ± 1.1 for PPMS (*p* < 0.001). The median time from MS onset to reach EDSS 4 was 14.0 years for EOMS, 8.0 years for IOMS, and 4.0 years for LOMS (*p* < 0.001). The median time form the disease onset to assignment of EDSS 6.0 was 24.5 years for EOMS, 12.0 years for IOMS, and 6.8 years for LOMS (*p* < 0.001). The time from the disease onset to attainment of EDSS 4 was 10.8 ± 8.0 years for RRMS and 5.0 ± 2.8 for the PPMS patients (*p* < 0.001). The time from onset of MS to the assignment of a score of 6 points on the EDSS scale was 13.9 ± 6.8 years for the RRMS and 8.0 ± 5.1 for the PPMS patients (*p* < 0.001). Figure 1, Figure 2 and Figure 3 show the distribution of the time to disability equal to 4 and 6 points on the EDSS scale for the whole studied group.

After 10 years of the disease, 40% of the patients reached 4 points on the EDSS scale. The same percentage of the patients reached 6 points on the EDSS scale after 20 years from the disease onset. The patients’ gender significantly influenced the disability progression, and the observed difference was particularly visible after the first six years of the disease duration. The male patients reached disability corresponding to 4 and 6 points on the EDSS scale faster. The clinical variant of the disease also had a statistically significant influence on the disability progression in the studied patients. The patients with PPMS were the first to reach disability evaluated as 4 and 6 points on the EDSS scale, followed by SPMS and RRMS patients. 

Of the 133 patients with documented RRMS duration of at least two years, the average number of relapses was 2.1 ± 1.1 during that time. A weak statistically significant correlation was found between the number of relapses during the first two years of RRMS and the time to reach EDSS 4 (R = −0.23; *p* = 0.007). Of 123 RRMS patients with the disease duration of at least five years, the average number of relapses was 4.3 ± 2.4 during that time. A moderate statistically significant correlation was found between the number of relapses during the first five years of RRMS and the time to reach EDSS 4 (R = −0.33; *p* < 0.001). No statistically significant correlation was found between the number of relapses during the first two and five years of the disease and the time to reach EDSS 6. More than one disease relapse occurred in 130 RRMS patients. In this subgroup, the median of time to the second relapse was one year. A moderate statistically significant correlation was found between the first inter-relapse interval and the time to reach EDSS 4 (R = 0.42; *p* < 0.001) and EDSS 6 (R = 0.4; *p* = 0.005). In the group of the patients with relapsing onset MS, 34% experienced an incomplete remission of the first relapse. The median time to reach EDSS 4 was 11.0 years for the patients with complete recovery from the first relapse and 5.0 years for the patients with incomplete remission of the first relapse (*p* < 0.001). The mean time to EDSS 6 was 16.5 ± 6.8 years in the group with relapsing-remitting onset MS, with complete remission of the symptoms of the first relapse, and 10 ± 4.8 years in the patients with residual symptoms of the first MS relapse (*p* < 0.001).

Amongst the patients with relapsing onset MS treated with IMDs, no statistically significant difference in time to EDSS 4 (*p* = 0.202) and EDSS 6 (*p* = 0.597) was found.

## 4. Discussion

In our population of 375 Polish MS patients, gender distribution, age of disease onset, nature of first symptoms, and the distribution of clinical types of the disease were consistent with the globally accepted pattern for the multiple sclerosis course. We observed that there were over twice as many women as men, and this is consistent with the majority of European studies conducted to date [21,22,23].

Overall, approximately 82.0% of the MS population experienced mild or moderate disability (EDSS below 6.0). In the investigated group, the distribution of individual clinical multiple sclerosis variants was consistent with the data presented by Confavreux and Vukusic (RRMS—58%, SPMS—27%, PPMS—15%) and the New York State Multiple Sclerosis Consortium (RRMS—55%, SPMS—31%, PPMS—14%) [6,24,25]. In our study, the relapsing-remitting onset of the disease was observed in 84.8% and the primary progressive onset was noted in 16.2% of the patients. When the progressive-relapsing multiple sclerosis (PRMS) is considered as the subgroup of PPMS, the quoted data is consistent with the generally accepted pattern for the initial MS course [15,21]. In our study, the primary progressive disease was statistically more frequently noted in the group of men than in women (25.2% vs. 10.8%). In contrast, in the group studied by Alonso et al. [25], no influence of the gender on the PPMS morbidity ratio was found. This discrepancy may also be explained by PRMS being distinguished by some authors as a separate clinical variant of the disease.

In the study cohort, the mean age at which first MS manifestations occurred corresponded with the data presented by Termelett et al. (32.4 ± 10.3 years) and Debouverie et al. (33 ± 10 years), and was higher than in the group of Kułakowska et al. (30.4 ± 9.8 years) and Brola et al. (30.8 ± 9.8 years) [9,15,23,26]. In our study, the percentage of EOMS patients was within the range presented by Chitnis et al., of 2–5%, with each person having had the relapsing-remitting disease onset [27]. The mean age of the first MS manifestations—PPMS onset later by 10 years—and the clinical course of early- and late-onset forms observed in this study were consistent with the data presented by Scalfari et al. [28].

In the majority of reports that evaluate the MS course published to date, the monosymptomatic disease onset predominated, and most frequently, its first symptom was sensory loss. In our study, when all patients were evaluated, despite the predominating monosymptomatic disease onset, the first clinical manifestation was usually motor deficit, followed by sensory symptoms and optic neuritis. In this context, the data presented in the Atlas of MS are worth quoting, where the most common initial MS symptom was sensory loss (40%). However, motor disorders as the first clinical manifestation were found in is as many as 39% of the patients [21]. In some of the available reports evaluating the cohort of Polish MS patients, a distribution of the number and the nature of first symptoms was found to be similar to our results, and this may indicate a slightly different initial disease course in this population. In the study by Brola et al., the disease onset was also usually monosymptomatic (78.4%) and had the form of a motor deficit (34.2%). It was followed by optic neuritis and sensory disturbances found in 25.2% and 18.3% of the patients, respectively [26]. In the group of Pierzchała et al., the most common initial MS symptoms were also motor deficits found in 50.3% of patients, followed by balance disorders (48%), double vision (40.1%), and numbness (23.9%) [29].

In our study, the nature of the first MS symptoms depended on the clinical variant of the disease. Amongst the RRMS patients, the first symptoms were most commonly manifested as sensory disturbances (36.2%), followed by optic neuritis (29.3%), and motor deficits (28%). The obtained results were similar to the distribution of initial symptoms in the RRMS patients in a population studied by Confavreux and Vukusic [7]. Primary progressive MS (PPMS) was associated with significantly predominating motor deficits (77.2%) as the first clinical manifestation, and this was consistent with a generally accepted pattern for this variant of the disease [30].

In the natural course of the disease, RRMS conversion to SPMS is observed between 5.8 and 19.1 years from the occurrence of first symptoms. The most likely median for this time is ca. 19 years [6,31,32]. In accordance with the Lublin and Reingold consensus of 1996, this variant is diagnosed in patients with relapsing-remitting onset, after confirming the progression of neurological deficits for a period of at least six months, regardless of the relapse occurrence [1]. In some of the previous studies, the observation time for the progressing neurological deficit was, however, 12 months, and the developed proposal for new SPMS diagnostic criteria takes into account only three-month observation, assuming a minimum patient disability corresponding to 4 points on the EDSS scale [33].

The wide span for the time of RRMS conversion to SPMS, as well as problems with comparing that parameter between the studies, result mainly from a lack of a specific biomarker and/or uniform criteria for diagnosing SPMS onset. Frequently, the onset of secondary MS occurs when a disability equal to 4 points on the EDSS scale is reached. Further observation of patients until they reach the disability of EDSS 6 allows for a more precise determination of that moment [34].

In our group, the mean time for RRMS conversion to SPMS was comparable to results obtained in the group of Eriksson et al., for patients with clinically possible or confirmed MS (12 ± 1.8 years), and in Potemkowski’s group (11.3 ± 4.2 years) [32,35]. In the study by Sand et al., the mean time for RRMS conversion to SPMS was 16.7 ± 2.0 years [36]. In that study, the mean time of a delay in diagnosing this variant of MS was also evaluated to be 2.9 ± 0.8 years. According to other authors, the delay observed could result from the fear to diagnose another stage of the disease, from some doctors treating this form as a failure of previous immunomodulating treatment and a need to discontinue it, and a lack of effective pharmacotherapy for this MS variant [37]. In our group, the mean time of RRMS conversion to SPMS was longer than the mean time to EDSS 4, and shorter than the mean time to EDSS 6. Faster conversion to SPMS was associated with the polysymptomatic onset, male gender, and initial MS symptoms in the form of motor deficits, balance disorders, motor coordination disorders, and sphincter dysfunctions. The statistical significance was noted solely for the polysymptomatic disease onset, and this was consistent with the data reported by Eriksson et al., and for initial motor symptoms of MS, and this, in turn, was consistent with the data obtained by Confavreux et al. [6,32]. In our study, of all factors delaying RRMS conversion to SPMS, statistical significance was only achieved for initial symptoms in the form of optic neuritis, and this observation also confirms the earlier reports of Confavreux et al. [6]. It is worth noting that in Poland the diagnosis of SPMS requires termination of IMDs, which could lead to an underestimation of the number of patients with this MS variant in the presented study.

In our group, PPMS patients reached disability corresponding to 4 and 6 points on the EDSS scale ca. six years earlier, on average, than the patients with the relapsing-remitting initial course of MS. After five years of the disease, the mean EDSS value in the patients with the primary progressive form of the disease was nearly twice as high as in RRMS patients, and amounted to 4.1 ± 1.1. The mean time to EDSS 4 for PPMS patients was half as long as for RRMS patients and the time to EDSS 6 in the subgroup of PPMS patients was similar to the results obtained by Cottrell et al. [38]. The factors increasing the risk of reaching both EDSS 4 and EDSS 6 in the whole group included longer disease duration, an older age of MS onset, the primary progressive disease course, and male gender. The relationships found were consistent with the generally accepted pattern for the MS course, and earlier studies conducted by Confavreux and Vukusic, and Eriksson et al., among others [6,32,39].

Amongst the patients with relapsing onset MS, the higher number of relapses during the first two and five years of treatment was correlated with the shorter time to EDSS 4; however, we did not demonstrate a correlation between that parameter and the time to EDSS 6.0. Many previous studies confirm an adverse influence of the larger number of relapses during the first two and five years of the disease on the disability progress. In the study by Scafrani et al. [10], it was found that the number of relapses during the first two years of the disease influenced the time to reach EDSS scores of 6, 8, and 10. In our study, the longer interval between the first and the second relapse was correlated with a longer time to disability equal to 4 and 6 points on the EDSS scale. The presence of residual symptoms of the first relapse statistically significantly shortened the time to EDSS 4 and 6. In their systematic review, Langer-Gould et al. [40] found that the greater frequency of relapses during the first years of the disease was not always correlated with a poor prognosis, and predictors for the faster progression of disability were initial disease symptoms in the form of sphincter dysfunctions, incomplete remission of symptoms of the first relapse, and a short time between the first and the second relapse. Thus, a thorough analysis of the recovery from the first MS relapse and determination of the time between first two relapses seems to be of greater importance than the total number of relapses in the first two and five years of the disease in predicting long-term disability progression in the patients.

In RRMS patients, there was no influence of treatment with IMDs on the time to reach 4 and 6 points on the EDSS scale. The obtained results do not form any grounds for conclusions concerning IMDs efficacy due to the short period of their use and the small percentage of the treated patients. The compared groups of the treated and untreated patients in our study were not homogeneous in terms of disease, clinical and radiological activity, and were partly a consequence of administrative limitations concerning the use of IMDs in Poland before 2014. In several previous studies describing the natural history of multiple sclerosis, a certain percentage of patients treated with immunosuppressive or immunomodulating drugs was found. They represented 46 of 216 subjects in the group of Cottrell et al. [38], and 903 of 1844 subjects in the group of Confavreux et al. [6]. In both studies, a significant influence of the treatment used on the evaluated parameters was excluded because the time of its use was too short, or there was no influence on the disability accrual. In this context, our study may also be considered as an analysis of the natural history of MS in the cohort of Polish patients.

The main limitation of this study was its retrospective nature and a relatively small group of subjects. Another limitation could be the ratio of patients receiving IMDs (33.3%) as mentioned above. The study was limited by the lack of information on the neuroradiological assessment of the disease. Data on the number, distribution, and contrast enhancement of demyelinating lesions in the initial magnetic resonance imaging (MRI) were available for half of the patients with a large variation of MRI scanners (from 0.5 to 1.5 T) on which they were performed. For this reason, we did not take into account the neuroradiological evaluation of the disease. In the study group, routine assessment of cognitive functions was not carried out. The lack of analysis of this parameter in relation to disease progression was also a limitation of our work.

## 5. Conclusions

Our results emphasize the importance of early clinical MS parameters in determining the clinical disease variant and the time to conversion from RRMS to SPMS as well as predicting the rate of disability accrual in patients. In the age of general availability of disease-modifying therapy, their analysis is of particular importance for identifying patients requiring more aggressive treatment, as well as for a selection of a proper treatment. In the present study, data regarding the long-term disability outcomes of Polish MS patients remain consistent with results from other regions of the world in the vast majority of the analyzed parameters.

## Figures and Tables

**Table 1:** Demographic and clinical characteristics of multiple sclerosis (MS) patients.

Characteristics of the Study Population	Total MS Patients	Female	Male
All patients	375	260 (69.3%)	115 (30.7%)
Mean age (years)	43.1 ± 12.5	42.2 ± 12.6	44.9 ± 12.0
Median time of disease duration (years)	9.0	8.0	9.0
Mean age at the first clinical manifestation of disease (years)	32.3	31.7 ± 10.9	33.5 ± 10,9
**Mean Expanded Disability Status Scale (EDSS) Score at Censoring (1–8)**	3.7 ± 1.7	3.5 ± 1.7	4.3 ± 1.7
0–3.5	188 (50.1%)	143 (55%)	45 (39.1%)
4.0–5.5	120 (32.0%)	80 (30.8%)	40 (34.8%)
6.0–10.0	67 (17.9%)	37 (14.2%)	30 (26.1%)
**Clinical Course at Censoring**			
Relapsing-remitting MS (RRMS)	214 (57.1%)	166 (63.8%)	48 (41.7%)
Primary progressive MS (PPMS)	57 (15.2%)	28 (10.8%)	29 (25.2%)
Secondary progressive MS (SPMS)	104 (27.7%)	66 (25.4%)	38 (33.1%)
**Localization of the First Demyelinating Lesions**			
Supratentorial structures and optic nerves	138 (64.8%)		
Spinal cord	132 (35.2%)		
Cerebellum	60 (16.2%)		
Brain stem	45 (12.0%)		
**Nature of Initial Symptoms**			
Motor deficit	133 (35.5%)		
Sensory symptoms	126 (33.6%)		
Optic neuritis	93 (24.8%)		
Balance disturbances	74 (19.7%)		
Vertigo	41 (10.9%)		
Double vision	35 (9.3%)		
Cranial nerves dysfunction	22 (5.9%)		
Sphincter dysfunctions	13 (3.5%)		
Gait dysfunctions	8 (2.1%)		
Coordination impairment	7 (1.9%)		
Neuralgias	6 (1.6%)		
Monosymptomatic onset	240 (64%)		
Polysymptomatic onset	135 (36%)		
**Treatment**			
Immunomodulatory drugs IMDs	125 (33.3%)		

**Table 2:** Initial MS symptoms depending on the clinical disease course.

Symptoms	PPMS n = 57		RRMS n = 318		*p*
	*n*	%	*n*	%	
Motor deficits	44	77.2%	89	28.0%	<0.001
Sensory loss	11	19.3%	115	36.2%	0.013
Optic neuritis	0	0.0%	93	29.3%	<0.001
Balance disturbances	21	36.8%	53	16.7%	<0.001
Vertigo	9	15.8%	32	10.1%	0.202
Double vision	2	3.5%	33	10.4%	0.100
Cranial nerves dysfunctions	7	12.3%	15	4.7%	0.025
Sphincter dysfunctions	5	8.8%	8	2.5%	0.017
Gait dysfunctions	4	7.0%	4	1.3%	0.006
Coordination impairment	1	1.8%	6	1.9%	0.940
Neuralgias	0	0.0%	6	1.9%	0.296

PPMS—primary progressive multiple sclerosis; RRMS—relapsing-remitting multiple sclerosis.

**Table 3:** The time of relapsing-remitting multiple sclerosis (RRMS) conversion to secondary progressive multiple sclerosis (SPMS), depending on the nature of the first symptom.

Symptoms	Conversion from RRMS to SPMS (years), n = 103				*p*
	Present Symptoms		Absent Symptoms		
	*n*	Mean ± SD	*n*	Mean ± SD	
Motor deficits	38	8.3 ± 4.4	65	15.3 ± 7.6	<0.001
Sensory loss	34	13.5 ± 8.0	69	11.5 ± 5.9	0.233
Optic neuritis	24	16.8 ± 5.2	79	11.5 ± 7.6	0.002
Balance disturbances	25	11.2 ± 6,8	78	13.2 ± 7.6	0.230
Vertigo	14	14.6 ± 11.7	89	12,4 ± 6.6	0.299
Double vision	8	13.3 ± 11.5	95	12.7 ± 7.1	0.834
Cranial nerves dysfunctions	5	13.2 ± 2.8	98	12.7 ± 7.6	0.883
Sphincter dysfunctions	2	8.5 ± 2.1	101	12.8 ± 7.5	0.420
Coordination impairment	2	5.5 ± 2.1	101	12.9 ± 7.4	0.167

## References

[1] Lublin F.D., Reingold S.C. (1996). Defining the clinical course of multiple sclerosis: Results of an international survey. National Multiple Sclerosis Society (USA) Advisory Committee on Clinical Trials of New Agents in Multiple Sclerosis. Neurology.

[2] Lassmann H., Brück W., Lucchinetti C.F. (2007). The immunopathology of multiple sclerosis: An overview. Brain Pathol..

[3] Sadovnick A.D., Ebers G.C. (1993). Epidemiology of multiple sclerosis: A critical overview. Can. J. Neurol. Sci..

[4] Leray E., Yaouanq J., Le Page E., Coustans M., Laplaud D., Oger J., Edan G. (2010). Evidence for a two-stage disability progression in multiple sclerosis. Brain.

[5] Weinshenker B.G., Bass B., Rice G.P., Noseworthy J., Carriere W., Baskerville J., Ebers G.C. (1989). The natural history of multiple sclerosis: A geographically based study: I: Clinical course and disability. Brain.

[6] Confavreux C., Vukusic S., Adeleine P. (2003). Early clinical predictors and progression of irreversible disability in multiple sclerosis: An amnesic process. Brain.

[7] Confavreux C., Vukusic S. (2006). Natural history of multiple sclerosis: A unifying concept. Brain.

[8] Tremlett H., Paty D., Devonshire V. (2006). Disability progression in multiple sclerosis is slower than previously reported. Neurology.

[9] Tremlett H., Zhao Y., Devonshire V. (2008). Natural history of secondary-progressive multiple sclerosis. Mult. Scler..

[10] Scalfari A., Neuhaus A., Degenhardt A., Rice G.P., Muraro P.A., Daumer M., Ebers G.C. (2010). The natural history of multiple sclerosis: a geographically based study 10: Relapses and long-term disability. Brain.

[11] Kremenchutzky M., Rice G.P., Baskerville J., Wingerchuk D.M., Ebers G.C. (2006). The natural history of multiple sclerosis: A geographically bases study. 9: Observations on the progressive phase of the disease. Brain.

[12] Tutuncu M., Tang J., Zeid N.A., Kale N., Crusan D.J., Atkinson E.J., Siva A., Pittock S.J., Pirko I., Keegan B.M. (2013). Onset of progressive phase is an age-dependent clinical milestone in multiple sclerosis. Mult. Scler..

[13] Confavreux C., Aimard G., Devic M. (1980). Course and prognosis of multiple sclerosis assessed by the computerized data processing of 349 patients. Brain.

[14] Kantarci O., Siva A., Eraksoy M., Karabudak R., Sütlaş N., Ağaoğlu J., Turan F., Ozmenoğlu M., Toğrul E., Demirkiran M. (1998). Survival and predictors of disability in Turkish MS patients. Turkish Multiple Sclerosis Study Group (TUMSSG). Neurology.

[15] Debouverie M., Pittion-Vouyovitch S., Louis S., Guillemin F., LORSEP Group (2008). Natural history of multiple sclerosis in a population-based cohort. Eur. J. Neurol..

[16] Poser C.M., Paty D.W., Scheinberg L., McDonald W.I., Davis F.A., Ebers G.C., Johnson K.P., Sibley W.A., Silberberg D.H., Tourtellotte W.W. (1983). New diagnostic criteria for multiple sclerosis: Guidelines for research protocols. Ann. Neurol..

[17] McDonald W.I., Compston A., Edan G., Goodkin D., Hartung H.P., Lublin F.D., McFarland H.F., Paty D.W., Polman C.H., Reingold S.C. (2001). Recommended diagnostic criteria for multiple sclerosis: Guidelines from the International Panel on the diagnosis of multiple sclerosis. Ann. Neurol..

[18] Polman C.H., Reingold S.C., Edan G., Filippi M., Hartung H.P., Kappos L., Lublin F.D., Metz L.M., McFarland H.F., O’Connor P.W. (2005). Diagnostic criteria for multiple sclerosis: 2005 revisions to the "McDonald Criteria". Ann. Neurol..

[19] Polman C.H., Reingold S.C., Banwell B., Clanet M., Cohen J.A., Filippi M., Fujihara K., Havrdova E., Hutchinson M., Kappos L. (2011). Diagnostic criteria for multiple sclerosis: 2010 revisions to the McDonald criteria. Ann. Neurol..

[20] Kurtzke J.F. (1983). Rating neurologic impairment in multiple sclerosis: An expanded disability status scale (EDSS). Neurology.

[21] Multiple Sclerosis International Federation (MSIF) Atlas of MS 2013: Mapping Multiple Sclerosis Around the World. www.msif.org/wp-content/uploads/2014/09/Atlas-of-MS.pdf.

[22] Pugliatti M., Rosati G., Carton H., Riise T., Drulovic J., Vécsei L., Milanov I. (2006). The epidemiology of multiple sclerosis in Europe. Eur. J. Neurol..

[23] Kułakowska A., Bartosik-Psujek H., Hożejowski R., Mitosek-Szewczyk K., Drozdowski W., Stelmasiak Z. (2010). Selected aspects of the epidemiology of multiple sclerosis in Poland - A multicentre pilot study. Neurol. Neurochir. Pol..

[24] Jacobs L.D., Wende K.E., Brownscheidle C.M., Apatoff B., Coyle P.K., Goodman A., Gottesman M.H., Granger C.V., Greenberg S.J., Herbert J. (1999). A profile of multiple sclerosis: The New York State Multiple Sclerosis Consortium. Mult. Scler..

[25] Alonso A., Jick S.S., Olek M.J., Hernán M.A. (2007). Incidence of multiple sclerosis in the United Kingdom: Findings from a population-based cohort. J. Neurol..

[26] Brola W., Fudala M., Flaga S., Ryglewicz D., Potemkowski A. (2015). Polski rejestr chorych na stwardnienie rozsiane – Stan obecny, perspektywy i problemy. Aktualn. Neurol..

[27] Chitnis T., Glanz B., Jaffin S., Healy B. (2009). Demographics of pediatric-onset multiple sclerosis in an MS center population from the Northeastern United States. Mult. Scler..

[28] Scalfari A., Knappertz V., Cutter G., Goodin D.S., Ashton R., Ebers G.C. (2013). Mortality in patients with multiple sclerosis. Neurology.

[29] Pierzchala K., Adamczyk-Sowa M., Dobrakowski P., Kubicka-Baczyk K., Niedziela N., Sowa P. (2015). Demographic characteristics of MS patients in Poland’s upper Silesia region. Int. J. Neurosci..

[30] Rose A.S., Kuzma J.W., Kurtzke J.F., Namerow N.S., Sibley W.A., Tourtellotte W.W. (1970). Cooperative study in the evaluation of therapy in multiple sclerosis. ACTH vs. placebo – Final report. Neurology.

[31] Amato M.P., Ponziani G. (2000). A prospective study on the prognosis of multiple sclerosis. Neurol. Sci..

[32] Eriksson M., Andersen O., Runmarker B. (2003). Long-term follow up of patients with clinically isolated syndromes, relapsing-remitting and secondary progressive multiple sclerosis. Mult. Scler..

[33] Lorscheider J., Buzzard K., Jokubaitis V., Spelman T., Havrdova E., Horakova D., Trojano M., Izquierdo G., Girard M., Duquette P. (2016). Defining secondary progressive multiple sclerosis. Brain.

[34] Tedeholm H., Lycke J., Skoog B., Lisovskaja V., Hillert J., Dahle C., Fagius J., Fredrikson S., Landtblom A.M., Malmeström C. (2013). Time to secondary progression in patients with multiple sclerosis who were treated with first generation immunomodulating drugs. Mult. Scler..

[35] Potemkowski A. (1999). Epidemiologiczne badania czasu trwania choroby i długości życia chorych na stwardnienie rozsiane. Zdrow. Publiczne.

[36] Sand I.K., Krieger S., Farrell C., Miller A.E. (2014). Diagnostic uncertainty during the transition to secondary progressive multiple sclerosis. Mult. Scler..

[37] Rovaris M., Confavreux C., Furlan R., Kappos L., Comi G., Filippi M. (2006). Secondary progressive multiple sclerosis: Current knowledge and future challenges. Lancet Neurol..

[38] Cottrell D.A., Kremenchutzky M., Rice G.P., Koopman W.J., Hader W., Baskerville J., Ebers G.C. (1999). The natural history of multiple sclerosis: A geographically based study. The clinical features and natural history of primary progressive multiple sclerosis. Brain.

[39] Vukusic S., Confavreux C. (2003). Prognostic factors for progression of disability in the secondary progressive phase of multiple sclerosis. J. Neurol. Sci..

[40] Langer-Gould A., Popat R.A., Huang S.M., Cobb K., Fontoura P., Gould M.K., Nelson L.M. (2006). Clinical and demographic predictors of long-term disability in patients with relapsing-remitting multiple sclerosis: A systematic review. Arch. Neurol..

